# Laser Interventions for Intraoral Halitosis: A Systematic Review of Randomized Controlled Trials

**DOI:** 10.3390/pharmaceutics17081046

**Published:** 2025-08-12

**Authors:** Rafał Wiench, Jakub Fiegler-Rudol, Natalia Zięba, Maciej Misiołek

**Affiliations:** 1 Department of Periodontal Diseases and Oral Mucosa Diseases, Faculty of Medical Sciences in Zabrze, Medical University of Silesia, 40-055 Katowice, Poland; 2 Department of Otorhinolaryngology and Laryngological Oncology in Zabrze, Medical University of Silesia, 41-800 Zabrze, Poland; nataliazieba@sum.edu.pl (N.Z.); maciej.misiolek@sum.edu.pl (M.M.)

**Keywords:** antimicrobial photodynamic therapy, diode laser, Er,Cr:YSGG laser, intraoral halitosis, laser therapy, oral malodor, oral microbiology, randomized controlled trials, systematic review, volatile sulfur compounds

## Abstract

**Background**: This systematic review evaluated the efficacy of laser therapies and antimicrobial photodynamic therapy (aPDT) for the treatment of intraoral halitosis by synthesizing randomized controlled trials. **Methods**: A comprehensive search of the PubMed, Embase, Scopus, and Cochrane Library databases identified 14 eligible RCTs. **Results**: Laser-based interventions, including diode lasers and Er,Cr:YSGG lasers, and aPDT using photosensitizers such as methylene blue, toluidine blue, and *Bixa orellana* (annatto), effectively reduced volatile sulfur compound levels and associated bacterial populations compared to traditional methods like tongue scraping and antiseptic rinses. Combination treatments consistently demonstrated superior short-term efficacy, although treatment outcomes often declined after 7–14 days, indicating the necessity for repeated sessions or adjunctive oral hygiene measures. **Conclusions**: Methodological heterogeneity across studies regarding laser parameters, photosensitizer types, and outcome measurements highlighted the need for standardized protocols. Future research should focus on multicenter trials with extended follow-up and standardized microbiological evaluations to further validate these promising treatments and integrate them effectively into clinical practice.

## 1. Introduction

Intraoral halitosis, or oral malodor, is a multifactorial condition with high global prevalence, frequently cited as the third most common reason for seeking dental care after caries and periodontal disease [1,2,3]. It is characterized by an unpleasant odor emanating from the oral cavity, primarily resulting from the microbial degradation of proteins and peptides into volatile sulfur compounds (VSCs) such as hydrogen sulfide (H_2_S), methyl mercaptan (CH_3_SH), and dimethyl sulfide ((CH_3_)_2_S) [4,5]. These compounds are produced by anaerobic, proteolytic bacteria residing predominantly on the posterior dorsum of the tongue and in periodontal pockets, particularly in patients with poor oral hygiene or periodontal pathology [6,7,8]. Commonly implicated species include *Fusobacterium nucleatum*, *Porphyromonas gingivalis*, *Prevotella intermedia*, and *Solobacterium moorei* [9,10,11,12,13]. Traditional halitosis management focuses on mechanical debridement using tongue scrapers, tooth brushing, and antiseptic mouthwashes, including chlorhexidine and essential oils [14,15,16]. However, these approaches have several limitations [17]. Chlorhexidine, while effective, is associated with adverse effects such as tooth staining, altered taste sensation, and mucosal irritation, limiting long-term compliance. Moreover, mechanical methods may fail to eliminate bacteria embedded deep within the tongue papillae or biofilm structures [18,19,20,21,22]. These limitations underscore the need for alternative approaches that offer deeper tissue penetration, enhanced antimicrobial specificity, and fewer side effects [23,24,25]. Antimicrobial photodynamic therapy (aPDT) and laser-based interventions have emerged as innovative, minimally invasive modalities capable of targeting halitosis-associated pathogens with high specificity and minimal collateral tissue damage [26,27].

Photodynamic therapy involves three key components: (1) a photosensitizer that selectively binds to microbial cells, (2) a light source of an appropriate wavelength to activate the photosensitizer, and (3) molecular oxygen present in tissues. Upon light activation, the photosensitizer transitions to an excited state and undergoes energy transfer reactions, generating ROS, such as singlet oxygen (^1^O_2_) and hydroxyl radicals [28,29,30,31]. These ROS induce oxidative damage to microbial membranes, proteins, and DNA, leading to rapid cell death without promoting antibiotic resistance [32].

Photosensitizers commonly used in halitosis-related aPDT include methylene blue, toluidine blue O, indocyanine green, and natural pigments like Bixa orellana. The diode laser (typically 660 nm) or blue LED systems serve as the light source due to their compatibility with these agents and suitable tissue penetration depth [33]. Laser therapy, independent of photosensitizers, can also be employed for halitosis reduction. Photobiomodulation and mid-infrared lasers such as Er,Cr:YSGG (2780 nm) function through direct bacterial ablation, vaporization of the tongue biofilm, or photothermal effects that denature microbial enzymes critical for sulfur compound production [34]. Additionally, photobiomodulation may modulate local immune responses and reduce inflammatory markers, thereby limiting microbial recolonization. Digital tongue analysis post-laser application has demonstrated reductions in tongue coating and VSC levels, confirming its functional and aesthetic benefits [35,36]. The rationale for exploring these techniques in intraoral halitosis lies not only in their microbiological efficacy but also in their potential to reduce patient discomfort, improve compliance, and minimize adverse reactions. Several randomized controlled trials have evaluated these interventions across varied populations and clinical settings, reporting reductions in organoleptic scores, Halimeter VSC readings, and halitosis-associated bacterial counts. However, the heterogeneity in treatment protocols, differing wavelengths, photosensitizers, power outputs, irradiation durations, and follow-up periods pose challenges to drawing generalized conclusions regarding their clinical utility [32,33,34,35,36]. Moreover, while some studies suggest that aPDT and laser therapy outperform mechanical debridement and chemical rinses in both short-term and intermediate follow-up periods, others report only modest or transient improvements. The biological persistence of halitosis also raises concerns about microbial recolonization and the need for repeated treatment sessions. There is a pressing need to consolidate findings from high-quality RCTs, critically appraise methodological consistency, and assess long-term safety and efficacy to guide evidence-based clinical decision making. Therefore, the present systematic review aims to synthesize current randomized controlled trial evidence evaluating the efficacy of laser-based treatments and aPDT for the management of intraoral halitosis. Specific objectives include assessing treatment outcomes based on objective measures such as volatile sulfur compound levels, microbial quantification, and patient-reported halitosis scores. By providing an integrated appraisal of laser and aPDT interventions, this review seeks to inform clinical practice, identify gaps in the current evidence base, and highlight avenues for future research in halitosis management. Unlike previous narrative or scoping reviews, this study applies a focused, RCT-based approach to assess the clinical effectiveness of laser and aPDT therapies specifically for intraoral halitosis, allowing for stronger evidence synthesis and critical appraisal.

## 2. Materials and Methods

### 2.1. Focused Question

This systematic review was structured using the PICO [37] (Population, Intervention, Comparison, Outcome) framework to formulate the research question: In individuals affected by intraoral halitosis (Population), do laser therapies or aPDT (Intervention) result in greater reductions in oral malodor and associated microbial load (Outcome), compared to conventional treatments, placebo, or no intervention (Comparison)?

### 2.2. Search Strategy

**Figure 1:**
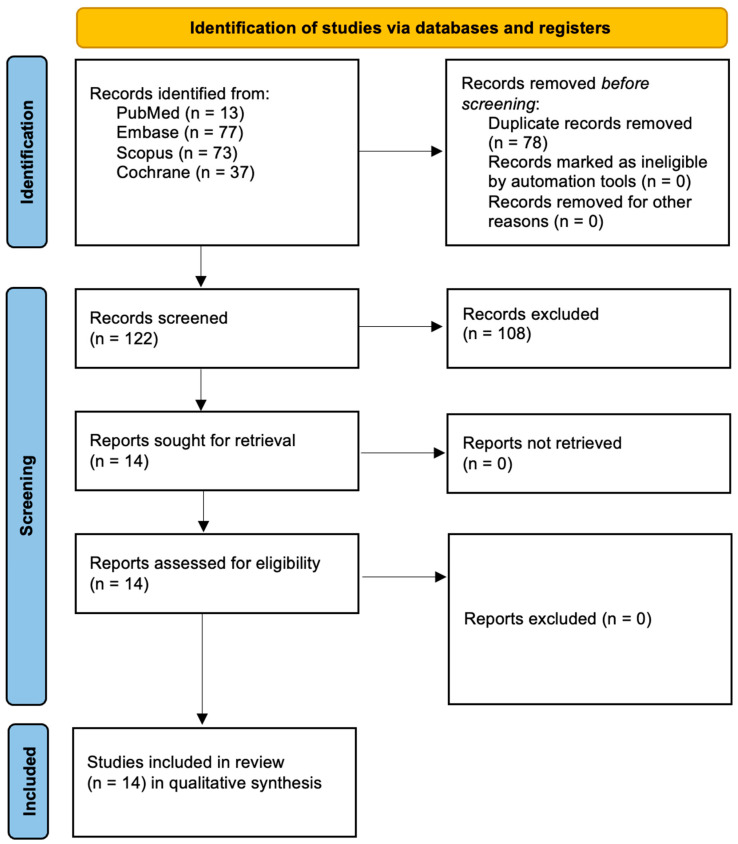
Prisma 2020 flow diagram.

This systematic review, registered in the PROSPERO database (Registration ID: CRD420251075670), was conducted in accordance with the PRISMA 2020 guidelines (Supplementary Materials) to ensure methodological transparency and scientific rigor [38]. A comprehensive literature search was carried out across four major electronic databases, PubMed/Medline, Embase, Scopus, and the Cochrane Library, to identify randomized controlled trials (RCTs) evaluating the efficacy of laser-based treatments and aPDT in managing intraoral halitosis. The complete search strategy is detailed in Figure 1. Three reviewers independently conducted the searches using a standardized set of keywords tailored to capture studies involving laser or aPDT interventions targeting halitosis. The inclusion criteria were limited to English-language publications, with no date restrictions. The study selection process involved an initial screening of titles and abstracts, followed by full-text evaluation by two independent reviewers based on predefined eligibility criteria (presented in Table 1). To ensure comprehensive identification of relevant trials, the reference lists of included studies were also manually searched for additional eligible RCTs.

### 2.3. Study Selection Process

To uphold methodological rigor and minimize the risk of bias, all identified studies were subjected to a structured, multi-phase screening process carried out independently by multiple reviewers. Initial screening of titles and abstracts was guided by predefined inclusion criteria. Any disagreements during the selection process were resolved through consensus-based discussion to ensure consistent and objective evaluation. The inclusion criteria were designed to capture high-quality randomized controlled trials examining the antimicrobial efficacy of laser-based interventions and aPDT in the treatment of intraoral halitosis. Eligible studies specifically assessed the effectiveness of laser or aPDT treatments, utilizing various photosensitizers, in reducing halitosis-associated microbial populations, with a focus on volatile sulfur compound-producing organisms. Trials employing rigorous designs with appropriate control groups, including placebo, no treatment, or alternative standard therapies, were considered. Only studies that clearly identified the targeted microorganisms and incorporated follow-up assessments to evaluate both the persistence of therapeutic effects and potential microbial recolonization were included. Studies were excluded if they lacked peer review, such as conference abstracts, case reports, editorials, opinion pieces, book chapters, or unpublished theses. Additional exclusion criteria included non-English publications, studies lacking scientific rigor, duplicate reports, or multiple publications derived from the same dataset without presenting new findings. Research not focused on intraoral halitosis or not involving infectious or microbial components relevant to halitosis was also excluded. Furthermore, studies without a control or comparison group were not considered. Trials that did not evaluate laser or aPDT as a primary intervention, those employing unrelated technologies, or studies focusing on non-relevant microbial targets were excluded. In vitro experiments conducted under artificial conditions that lacked translational or clinical relevance were similarly omitted.

### 2.4. Risk of Bias in Individual Studies

To ensure objectivity and minimize selection bias, the screening of titles and abstracts identified during the literature search was performed independently by multiple reviewers. Inter-rater reliability was evaluated using Cohen’s kappa statistic, providing a quantitative measure of agreement between reviewers [39]. Discrepancies between reviewers during the risk-of-bias assessment were resolved through consensus-based discussions. If consensus could not be reached, a third reviewer acted as an adjudicator to make the final decision. This systematic and transparent approach was implemented to strengthen the methodological integrity of the review and ensure the accurate identification of eligible randomized controlled trials investigating laser and aPDT interventions for intraoral halitosis.

### 2.5. Quality Assessment

The methodological quality of the included studies was independently evaluated by three reviewers. In this review, we used the Risk of Bias 2 (RoB 2) tool, developed by the Cochrane Collaboration, to assess the methodological quality of the included randomized controlled trials [40]. This tool evaluates the internal validity of each study by examining five specific domains: the randomization process, deviations from intended interventions, missing outcome data, measurement of the outcome, and selection of the reported result. Each domain was judged as having a low risk of bias, some concerns, or high risk of bias based on predefined signaling questions. An overall risk of bias judgment was then determined for each study. The use of RoB 2 provided a systematic and transparent approach to identifying potential sources of bias that could influence the reliability of the trial findings. Table 2 shows the results of the quality assessment.

Several studies in the review were rated as having “some concerns” in various RoB 2 domains due to insufficient reporting or potential methodological bias. In Alshahrani et al., 2020 [41] and Gonçalves et al., 2020 [47], randomization procedures were not clearly described, raising concerns about allocation concealment. Joseph et al., 2016 [42] involved subjective outcomes such as patient-reported halitosis without adequate information on assessor blinding, suggesting risk of bias in outcome measurement. Ciarcia et al., 2019 [44] and Krespi et al., 2021 [49] also lacked clarity regarding randomization methods, and the latter had high attrition without explanation, suggesting possible bias from missing data. Furthermore, do Vale et al., 2021 [46], Joseph et al., 2016 [42], Ciarcia et al., 2019 [44], Gonçalves et al., 2020 [47], and Krespi et al., 2021 [49] all lacked access to a pre-specified protocol or statistical analysis plan, making selective reporting a concern across these trials. These issues led to an overall risk of bias judgment of “some concerns” for those studies. The studies rated with “some concerns” in the second RoB 2 assessment set showed issues primarily related to insufficient reporting of randomization and lack of pre-specified protocols. Lopes et al., 2014 [50] and Pinto et al., 2016 [53] were both described as randomized but did not clearly explain the method of sequence generation or allocation concealment, leading to concerns about potential selection bias. Additionally, both lacked access to a published statistical analysis plan, raising the possibility of selective outcome reporting. Lopes et al., 2016 [50] had adequate randomization and outcome measurement procedures, but like the others, did not provide a pre-registered protocol or clearly indicate whether all pre-specified outcomes were reported, resulting in concerns about selective reporting. Overall, these deficiencies in transparency and reporting consistency led to “some concerns” ratings in the relevant RoB 2 domains.

### 2.6. Data Extraction

For each included study, data extraction was conducted independently by three reviewers using a predefined, standardized protocol to ensure accuracy and reproducibility. The reviewers collected detailed information including the first author, year of publication, study design, characteristics of the study population, intervention and control group descriptions, and the duration of follow-up. Emphasis was placed on parameters relevant to laser and aPDT, including the type and concentration of the photosensitizer, specifications of the light source (such as wavelength, power output, and energy density), method and frequency of application, and any adjunctive treatments used. In addition, primary and secondary outcomes related to the reduction in intraoral halitosis and microbial load were recorded, including objective measures such as volatile sulfur compound levels and microbial assays. Procedural details, such as irradiation technique, duration, and treatment sessions, were extracted to allow for meaningful comparison across studies. Where applicable, outcomes related to patient-reported measures, safety, and post-treatment recolonization were also documented. This structured approach allowed for comprehensive synthesis and facilitated analysis of the technical and clinical variables influencing treatment efficacy.

## 3. Results

### 3.1. Study Selection

In alignment with the PRISMA 2020 guidelines, the study selection process is illustrated in Figure 1. A total of 200 records were initially identified through database searches, including PubMed (*n* = 13), Embase (*n* = 77), Scopus (*n* = 73), and Cochrane Library (*n* = 37). After the removal of 78 duplicate records, 122 unique articles remained for title and abstract screening. Of these, 108 records were excluded based on predefined eligibility criteria, leaving 14 full-text reports assessed for eligibility. No reports were excluded or not retrieved at this stage. Ultimately, all 14 studies met the inclusion criteria and were included in the qualitative synthesis. These randomized controlled trials formed the evidence base for evaluating the efficacy of laser and aPDT interventions in the treatment of intraoral halitosis.

### 3.2. Data Presentation

Table 3, Table 4 and Table 5 provide a structured and comprehensive summary of the findings from the fifteen included randomized controlled trials, highlighting key clinical outcomes, methodological features, and microbiological evidence related to the antimicrobial effectiveness of laser and aPDT in the treatment of intraoral halitosis.

### 3.3. Overview of Study Characteristics

Table 3 presents an overview of the included studies.

**Table 3:** Summary of the origin and aims of the included studies.

Study	Geographic Location	Aim
Alshahrani et al., 2020 [41]	Saudi Arabia	Evaluate efficacy of PDT with diode laser and methylene blue in halitosis during orthodontic treatment.
Joseph et al., 2016 [42]	India	Assess aPDT as adjunct to SRP in chronic periodontitis.
Bruno et al., 2024 [43]	Italy	Compare efficacy of aPDT vs. conventional treatment for halitosis in adolescents.
Ciarcia et al., 2019 [44]	Italy	Test aPDT with erythrosine and red LED for halitosis in periodontitis patients.
Dereci et al., 2016 [45]	Turkey	Evaluate Er,Cr:YSGG laser-assisted periodontal therapy on halitosis and periodontal healing.
do Vale et al., 2021 [46]	Brazil	Compare aPDT and tongue scraping for halitosis in older adults with dentures.
Gonçalves et al., 2020 [47]	Brazil	Test aPDT with Bixa orellana extract and blue LED in halitosis reduction.
Joseph et al., 2014 [48]	United States	Assess laser tongue debridement for oral malodor reduction.
Krespi et al., 2021 [49]	Brazil	Evaluate aPDT alone and combined with scraping in adolescent halitosis treatment.
Lopes et al., 2014 [50]	Brazil	Compare effect of photodynamic therapy and tongue scraping on halitosis in adolescents using gas chromatography.
Lopes et al., 2016 [51]	Brazil	Evaluate antimicrobial effect of annatto-based PDT versus chlorhexidine and tongue scraper on halitosis in children.
da Mota et al., 2016 [52]	Brazil	Assess effects of SRP with or without PDT on halitosis and periodontal health in bronchiectasis patients.
Pinto et al., 2016 [53]	Brazil	Determine whether oral hygiene behavior combined with aPDT or tongue scraper reduces halitosis over 90 days.
Romero et al., 2021 [54]	Brazil	To verify whether modification of oral hygiene behavior associated with aPDT or a lingual scraper can reduce halitosis after a 90-day follow-up.

PDT—photodynamic therapy, aPDT—antimicrobial photodynamic therapy, SRP—scaling and root planing LED—Light Emitting Diode, Er,Cr:YSGG—Erbium, Chromium-doped Yttrium Scandium Gallium Garnet.

### 3.4. Main Study Outcomes

Table 4 presents the main outcomes of the study. For clarity, the clinical effects of each intervention have been grouped based on follow-up duration: immediate (≤24 h), short-term (up to 14 days), and intermediate term (approximately 30 days). This classification helps to distinguish temporary effects from more sustained therapeutic outcomes and enhances interpretability across studies.

**Table 4:** Summary of principal results and study details.

Author and Year	Study Groups	Outcomes
Alshahrani et al., 2020 [41]	1. (PDT only) 2. (TS only) 3. (PDT + TS)	The group receiving both PDT and TS achieved a 100% reduction in H_2_S levels after 2 weeks, significantly more than PDT or TS alone. Only the combined PDT + TS group showed a statistically significant reduction in all five targeted oral pathogens on the tongue (e.g., *P. gingivalis*, *T. denticola*). PDT used by itself reduced malodor comparably to mechanical scraping, but neither alone significantly reduced bacterial counts. The reduction in halitosis and bacterial load was effective over the short term, and long-term efficacy remains unproven and needs further investigation.
Joseph et al., 2016 [42]	1. (Control) Tongue scraping only 2. Tongue scraping followed by chlorhexidine mouthwash 3. (Experimental) Tongue scraping followed by aPDT using MB and a diode laser at 660 nm	Halitosis significantly improved at 1 month in the group receiving aPDT in addition to SRP, compared to SRP alone (*p* < 0.05). Reduction in bleeding gums was perceived earlier and more significantly in the aPDT group at 2 weeks and 1 month post-treatment (*p* < 0.05). Pain while chewing decreased significantly in the aPDT group at 2 weeks and 1 month compared to control (*p* < 0.05), aligning with clinical improvements. Patients reported short-term symptomatic relief from a single aPDT session, though no long-term differences or differences in treatment acceptance and sensitivity were observed
Bruno et al., 2024 [43]	1. (Experimental) aPDT using annatto-based photosensitizer and blue LED 2. (Control) Tongue scraping only	aPDT significantly reduced halitosis scores in mouth-breathing children compared to baseline and was more effective than mechanical tongue scraping at all post-treatment time points (immediately, 7 days, 30 days) (*p* < 0.0001). The treatment effect of aPDT was sustained, with no significant rebound in halitosis levels at 7 or 30 days post-intervention, indicating prolonged benefit. No correlation was found between coated tongue index and halitosis severity, suggesting that tongue coating was not a primary contributor to halitosis in this cohort. aPDT using annatto and blue LED was well tolerated, non-invasive, and potentially superior to standard tongue scraping, offering a viable clinical approach for managing halitosis in paediatric mouth-breathers.
Ciarcia et al., 2019 [44]	1. (Experimental) MB-aPDT and a 660 nm diode laser 2. (Control) Received a placebo treatment without laser activation	Immediate reduction in VSCs such as hydrogen sulfide and methylmercaptan was observed after a single aPDT session on the dorsum of the tongue, indicating direct deodorizing efficacy. Significant microbiological changes were measured, specifically a reduction in halitosis-associated anaerobic bacteria including *P. gingivalis*, *T. forsythia*, and *T. denticola*, as confirmed by qPCR analysis. The combined use of a tongue scraper with aPDT produced superior results compared to either treatment alone, supporting the synergistic effect of mechanical and photodynamic decontamination. aPDT was non-invasive, fast, and showed no adverse effects, offering a safe therapeutic alternative with minimal risk of bacterial resistance, as its mechanism involves oxidative stress via singlet oxygen and free radicals.
Dereci et al., 2016 [45]	1. (Experimental) aPDT using toluidine blue and a diode laser 2. (Control) Placebo treatment without laser irradiation	Er,Cr:YSGG laser-assisted periodontal therapy significantly reduced oral malodor compared to conventional therapy alone. Statistically significant reductions in VSCs were noted at the 3rd and 6th months post-treatment in the laser-treated group. Laser treatment also resulted in significant improvements in periodontal parameters, notably probing depth at 1 month and bleeding on probing at 3 and 6 months. The laser-assisted approach provided better periodontal healing and more effective control of halitosis associated with periodontal disease compared to traditional periodontal therapy.
do Vale et al., 2021 [46]	1. (Experimental) Treated with antimicrobial aPDT using a 660 nm laser and annatto-based photosensitizer 2. (Control) Placebo	Both organoleptic scores and Halimeter readings showed significant improvements following photodynamic therapy with methylene blue. aPDT was more effective in reducing intraoral halitosis compared to placebo and tongue brushing alone. The treatment led to a measurable decrease in halitosis-associated bacterial species, particularly those producing volatile sulfur compounds (VSCs). No adverse events were reported, indicating the safety and tolerability of the aPDT protocol used.
Gonçalves et al., 2020 [47]	1. aPDT with Bixa orellana extract and blue LED 2. (Control) Tongue scraping only 3. Tongue scraping and aPDT with Bixa orellana extract and blue LED	MB-aPDT significantly lowered VSC levels compared to the control group. aPDT demonstrated a greater reduction in halitosis scores than placebo treatment, confirming its therapeutic benefit. aPDT led to decreased levels of halitosis-associated bacteria, notably those responsible for sulfur compound production. The intervention was safe and well tolerated by participants, with no discomfort or complications observed.
Joseph et al., 2014 [48]	1. (Experimental) Tongue brushing followed by aPDT using methylene blue and a 660 nm diode laser 2. (Control) Tongue brushing only	MB-aPDT and a diode laser led to a statistically significant reduction in VSC compared to baseline. aPDT showed superior results in reducing halitosis compared to tongue scraping alone. The beneficial effects were sustained for several days post-treatment, supporting its short-term efficacy. The treatment was well tolerated, with no participants experiencing pain, irritation, or side effects.
Krespi et al., 2021 [49]	1. (Experimental) Treated with Er,Cr:YSGG laser tongue debridement. 2. (Control) Treated with tongue scraper, each initially comprising 30 patients	LTD using Er,Cr:YSGG significantly reduced oral malodor, as measured by both organoleptic scores and Halimeter readings, with sustained effects for at least one month post-treatment. The laser was more effective than tongue scraping in reducing both aerobic and anaerobic bacterial load on the tongue dorsum, demonstrating statistically significant CFU reductions (*p* = 0.010 for anaerobic and *p* = 0.002 for aerobic cultures immediately post-treatment). Patients reported minimal discomfort during LTD, and quality of life improvements were documented through HALT scores, which showed significant enhancement in the laser group compared to control (*p* = 0.013). Digital tongue color analysis showed increased healthy (pink) tones and decreased foul (brown) coatings following laser treatment, correlating with reductions in bacterial biofilm and volatile sulfur compounds (VSCs).
Lopes et al., 2014 [50]	1. (Experimental) MB-aPDT and a red diode laser after tongue scraping 2. (Control) Underwent tongue scraping only.	The study demonstrated that MB-based aPDT using a low-level laser led to a significant reduction in volatile sulfur compound levels in individuals with halitosis. The aPDT group showed greater halitosis reduction than the control and mechanical cleaning groups across all post-treatment time points. Reduction in halitosis was maintained through multiple follow-up assessments, indicating persistent antimicrobial and deodorizing effects. The procedure was well tolerated by participants, with no reports of pain, irritation, or other side effects, confirming its safety.
Lopes et al., 2016 [51]	1. aPDT on the dorsum of the tongue 2. Treatment with a tongue scraper 3. Combination of tongue scraper and photodynamic therapy.	PDT alone significantly reduced halitosis, with a median reduction in H_2_S levels by 88.6% in adolescents after a single session, demonstrating its standalone effectiveness in targeting VSC-producing bacteria on the tongue. PDT combined with tongue scraping resulted in complete elimination of halitosis, achieving a 100% reduction in H_2_S concentrations. This combination was significantly more effective than either method alone. PDT was non-invasive and less aggressive than mechanical scraping, avoiding damage to the lingual papillae, which can occur with conventional tongue cleaning methods. Methylene blue and red light effectively reached bacteria located in tongue papillae, especially after initial biofilm reduction by scraping, supporting a synergistic mechanism that enhances antimicrobial action.
da Mota et al., 2016 [52]	1. aPDT only 2. Tongue scraper only, 3. Both photodynamic therapy and a tongue scraper.	aPDT significantly reduced halitosis in adolescents, with a 97.6% median reduction in H_2_S levels immediately after treatment. The combination of aPDT and tongue scraping resulted in a 100% median reduction, outperforming tongue scraping alone. A 7% decrease in bacterial load in the group treated with aPDT alone, a 7% increase in the scraper-only group, and no change in the combined treatment group. aPDT was more effective in reducing microbial burden than mechanical cleaning. The halitosis-reducing effect of aPDT was immediate but not sustained, as H_2_S levels in all groups returned close to baseline seven days post-treatment, indicating the need for repeated or maintenance therapy. aPDT offers a non-mechanical, minimally invasive alternative to tongue scraping that avoids trauma to tongue tissue and may be especially useful for adolescents with poor compliance to mechanical hygiene methods.
Pinto et al., 2016 [53]	1. (Experimental) Treated with aPDT using methylene blue and laser 2. (Control) Treated with mechanical tongue scraping only.	aPDT significantly reduced H_2_S levels after a single application, confirming its effectiveness in treating halitosis in adolescents. The combination of aPDT with tongue scraping yielded the greatest halitosis reduction, outperforming either treatment alone in lowering volatile sulfur compound concentrations. aPDT alone demonstrated effective microbial reduction, targeting tongue biofilm without the mechanical trauma associated with scraping. The therapeutic effects of aPDT were transient, as halitosis levels rebounded one-week post-treatment, suggesting the need for repeated applications to maintain benefits.
Romero et al., 2021 [54]	1. (Experimental) Received aPDT with urucum photosensitizer and LED light 2. (Control) Received placebo treatment without active photosensitizer.	aPDT significantly reduced VSC immediately after treatment, demonstrating a clear antimicrobial effect on halitosis-causing bacteria. The combination of aPDT with tongue scraping produced superior results, achieving greater halitosis reduction than either method alone. Microbial analysis showed a notable reduction in anaerobic bacterial colonies on the tongue dorsum following aPDT application. No adverse effects reported.

PDT—photodynamic therapy, TS—Tongue Scraping, aPDT—antimicrobial photodynamic therapy, MB—Methylene Blue, LED—Light Emitting Diode, VSCs—volatile sulfur compounds, SRP—scaling and root planing, qPCR—Quantitative Polymerase Chain Reaction, Er,Cr:YSGG—Erbium, Chromium-doped Yttrium Scandium Gallium Garnet laser, LTD—Laser Tongue Debridement, CFU—Colony-Forming Unit, HALT—Halitosis Associated Life-quality Test, H_2_S—hydrogen sulfide.

Table 5 summarizes the follow-up durations and timing of outcome assessments across studies evaluating halitosis treatment interventions.

**Table 5:** Follow-up durations and timing of outcome.

Study	Follow-Up Duration	Key Outcome
Alshahrani et al. (2020) [41]	Immediate	A 100% reduction in H_2_S in PDT + scraper group after treatment
Gonçalves et al. (2020) [47]	Immediate	Significant VSC reduction immediately; not maintained at 7 days
Lopes et al. (2014) [50]	Immediate	Reduction in halitosis at 1 and 24 h after treatment
da Mota et al. (2016) [52]	Immediate	Significant VSC reduction; median VSC = 0 in PDT + scraper group
Romero et al. (2021) [54]	Immediate	H_2_S reduced in both aPDT and scraper groups (*p* = 0.0001)
do Vale et al. (2021) [46]	Short Term (7 days)	Halitosis levels remained socially acceptable at 7 days
Bruno et al. (2024) [43]	Short Term (7 days)	Sustained reduction in halitosis at 7 days post-treatment
Ciarcia et al. (2019) [44]	Short Term (7–14 days)	Reduction in VSC at 7 and 14 days, partially sustained
Romero et al. (2021) [54]	Short Term (7 days)	H_2_S 3x lower in aPDT and 2× lower in scraper group at 7 days
Joseph et al. (2014) [48]	Intermediate (30 days)	Significant halitosis reduction at 1 month, then declined
Bruno et al. (2024) [43]	Intermediate (30 days)	Reduction in halitosis maintained at 30 days
Romero et al. (2021) [54]	Intermediate (90 days)	H_2_S remained 2–3× lower; halitosis still above clinical threshold
Dereci et al. (2016) [45]	Long Term (3–6 months)	Sustained VSC reduction at 3 and 6 months
Pinto et al. (2016) [53]	Intermediate (3 months)	Planned follow-up at 3 months; outcomes pending (protocol study)

aPDT—antimicrobial photodynamic therapy, H_2_S—hydrogen sulfide, PDT—photodynamic therapy, VSC—volatile sulfur compounds.

### 3.5. Measuring Halitosis

Gas Chromatography (GC) stands as the gold standard due to its ability to individually quantify volatile sulfur compounds like H_2_S, CH_3_SH, and CH_3_SCH_3_. Studies using GC provide more precise and reproducible data, strengthening the validity of treatment outcomes [55,56]. Halimeter and portable monitors provide quantitative results but lack the specificity of GC. Their readings can be influenced by environmental factors and provide aggregated VSC levels rather than compound-specific data. This introduces some uncertainty in interpreting the microbial etiology and treatment effectiveness [56,57]. Organoleptic tests, while reflecting real-world human perception, are inherently subjective and prone to inter-examiner variability. Studies relying primarily on organoleptic scoring may have weaker evidence due to potential bias unless complemented by objective tools. Multimodal approaches (e.g., combining GC with microbiological analysis or patient-reported outcomes) provide a more holistic and balanced assessment of halitosis, allowing for stronger, multifactorial evidence. This is presented in Table 6.

### 3.6. Characteristics of Light Sources Used in PDT

Table 7 shows the parameters of lasers used in each study.

### 3.7. Reported Adverse Effects and Safety

Across the included studies, the overall incidence of adverse effects was low, and no serious intraoperative or postoperative complications were reported for either laser therapy or aPDT. Most studies explicitly stated that treatments were well tolerated, with participants experiencing no discomfort, mucosal damage, or hypersensitivity reactions. Antimicrobial photodynamic therapy, particularly with methylene blue or annatto-based photosensitizers, was consistently reported as safe. No studies described allergic reactions, tissue necrosis, or systemic toxicity. The most noted precaution was the need to remove excess photosensitizer before irradiation to avoid superficial staining. Laser therapy, including diode and Er,Cr:YSGG lasers, also demonstrated a favorable safety profile. When applied using correct parameters and cooling (in the case of Er,Cr:YSGG), no cases of tissue burns or damage to lingual papillae were reported. However, unlike aPDT, high-energy laser application may theoretically pose a greater risk of thermal damage if applied improperly, particularly without adequate operator training or appropriate power settings. Overall, both modalities are minimally invasive and well tolerated, but aPDT, especially with low-power diode lasers or LED systems, may present a slightly lower procedural risk due to its photochemical rather than photothermal mechanism. Operator experience and adherence to safety protocols (e.g., eye protection, controlled dosing) remain critical for minimizing complications in both approaches.

## 4. Discussion

### 4.1. Results in the Context of Other Evidence

Photodynamic therapy has emerged as an effective antimicrobial approach for intraoral halitosis, operating through a specific photochemical mechanism involving a photosensitizer, targeted light irradiation, and molecular oxygen [55,56,57,58,59,60]. Activation of the photosensitizer by light generates reactive oxygen species (ROS), notably singlet oxygen, leading to bacterial membrane damage and subsequent cell death [56,61]. This action is particularly efficient against anaerobic bacteria, primarily responsible for volatile sulfur compounds’ production, and importantly, it mitigates the risk of antimicrobial resistance development [57,62]. Methylene blue is the most widely studied photosensitizer in halitosis treatment, employed typically in concentrations between 0.005% and 0.05% [56,63]. Its optimal activation occurs at 660 nm, a wavelength commonly used in diode lasers. MB has demonstrated consistent efficacy and a favorable safety profile in multiple randomized controlled trials [58,61,63]. Alternative photosensitizers, including toluidine blue and natural derivatives such as *Bixa orellana* (annatto), activated by blue LED light (395–480 nm), offer additional practical advantages, including reduced costs and ease of integration with conventional dental photopolymerization equipment [64,65,66,67]. Standard diode laser protocols involve power settings of 100–400 mW, energy doses of 9–36 J per application point, and irradiation durations of 60–90 s per site [57,62,65]. Treatments typically encompass 4–6 points on the posterior tongue dorsum, maintaining approximately a 1 cm spacing, with pre-irradiation incubation times for the photosensitizer ranging from 2 to 5 min. Excess photosensitizer is removed by saline irrigation before laser application. Alternative high-power laser systems, such as the Er,Cr:YSGG laser operating at 2780 nm, offer direct photothermal ablation and biofilm removal, employing higher power settings (1–4 W) and pulse frequencies (30–40 Hz), supported by water-mediated cooling to ensure tissue safety [62]. Clinical studies consistently report immediate and substantial reductions in VSC concentrations post-treatment, with reported decreases of up to 60% [64,65]. Hydrogen sulfide levels notably decrease from baseline values exceeding 100 ppb to below 60 ppb immediately following treatment [63]. The combination of PDT with mechanical tongue scraping demonstrates superior efficacy compared to monotherapies, significantly enhancing bacterial elimination and prolonging therapeutic outcomes [65,67]. Microbiological analyses employing real-time PCR have confirmed significant reductions in key halitosis-associated pathogens such as *Porphyromonas gingivalis*, *Fusobacterium nucleatum*, and *Treponema denticola* following PDT [63,66]. The depth of photodynamic action (0.5–1.5 cm) surpasses conventional mechanical cleaning methods, effectively reaching deeper bacterial reservoirs. However, treatment durability typically ranges between 7 and 14 days, with single-session interventions showing diminishing effects thereafter [61,63]. Enhanced outcomes have been reported when combined with regular oral hygiene practices, highlighting the importance of patient education in maintaining treatment benefits [64]. Comparatively, PDT outperforms conventional mechanical debridement (tongue scraping) and achieves bacterial reductions akin to antimicrobial mouthwashes, but without the risk of antimicrobial resistance or tissue irritation [62,67]. Combination approaches incorporating mechanical and photodynamic treatments consistently yield superior outcomes, with recent explorations into probiotics suggesting potential additional benefits through competitive bacterial colonization [65,67]. Regarding safety, PDT presents an excellent profile with no serious adverse events reported across extensive clinical trials [58,61,62]. The selectivity and low concentrations of photosensitizers further underscore this favorable safety record. However, PDT is contraindicated in patients with photosensitizer allergies or porphyria, with pregnant or lactating women typically excluded as a precaution [58,61]. Standardized safety protocols, including appropriate eye protection, are critical. Cost-effectiveness is improved by innovations such as annatto-based photosensitizers compatible with existing dental LED equipment, significantly reducing treatment costs [66,67,68,69]. However, despite promising results, clinical implementation remains challenging due to variability in treatment protocols and the need for specialized training in photodynamic therapy principles [59,60,62]. Future research should prioritize protocol standardization, longer-term efficacy studies, and multicenter trials to establish robust clinical guidelines [60,62]. Additionally, exploring combination therapies, novel photosensitizers, and home-based PDT devices could further enhance clinical outcomes, cost-effectiveness, and patient accessibility [61,62]. While all included studies reported significant short-term reductions in halitosis parameters following either laser- or LED-mediated photodynamic therapy, the current evidence does not allow for a definitive comparison of the relative superiority between these light sources. Both diode lasers (typically operating at 660 nm) and blue LEDs (395–480 nm) demonstrated comparable antimicrobial effects when paired with compatible photosensitizers. However, due to methodological heterogeneity, including variations in power output, spot size, application technique, and follow-up duration, direct comparisons remain inconclusive. Therefore, it is currently not possible to state which modality is superior for clinical halitosis management. This underscores the need for standardized, head-to-head trials evaluating different light sources under uniform conditions. The safety profiles of both aPDT and laser therapy were highly favorable across the included RCTs, with no serious adverse events reported. While laser devices such as Er,Cr:YSGG carry a theoretical risk of thermal injury if misused, this was not observed in any included trial. Conversely, aPDT offers a more targeted, photochemical action with minimal heat generation, potentially making it safer in less experienced hands or pediatric settings.

### 4.2. Limitations of the Evidence

Several limitations should be acknowledged regarding the current evidence base supporting laser and aPDT interventions for intraoral halitosis. Primarily, despite the inclusion of RCTs, there remains considerable variability in study designs, particularly concerning differences in laser types, wavelengths, power outputs, treatment durations, photosensitizer selection, and application methods. This methodological heterogeneity impedes definitive conclusions regarding optimal treatment protocols and their universal applicability. Additionally, most included studies feature relatively short-term follow-up periods, limiting insights into the long-term sustainability and clinical effectiveness of these interventions. Another significant limitation is the inconsistent reporting of patient demographics and potential confounding factors such as oral hygiene practices, smoking status, or concurrent systemic health conditions, which could influence treatment outcomes. Furthermore, standardized microbiological assessment methods and outcome measures are lacking, resulting in difficulties in objectively comparing microbial efficacy across different studies. Lastly, the relatively small sample sizes typical of many included trials potentially limit the statistical power and generalizability of the findings. Addressing these limitations through more rigorous, standardized, large-scale, multicenter trials with extended follow-up periods would significantly enhance the evidence base for laser and aPDT interventions in managing intraoral halitosis. Another underreported yet potentially important variable is the size of the light source or irradiation spot. Only a minority of studies included in this review explicitly stated beam diameter or spot size, limiting the ability to evaluate its influence on treatment outcomes. Given the possible relationship between beam size, power density, and uniform tissue coverage, future studies should systematically report and compare irradiation areas to optimize protocol efficiency and microbial reduction. When interpreting efficacy, it is critical to consider the follow-up duration. Several studies demonstrated substantial immediate or short-term improvements (within 1–14 days), but in many cases, the therapeutic effects diminished by 30 days post-treatment. The variability in follow-up durations among included RCTs underscores the need for standardized time points in future studies to better assess treatment sustainability. Furthermore, most included studies did not adequately control for key confounding variables such as oral hygiene practices, smoking status, salivary flow rate, systemic health conditions, or prior antimicrobial use. These uncontrolled factors may influence baseline halitosis severity and individual treatment responses, thereby limiting the internal validity and comparability of outcomes across studies.

### 4.3. Limitations of the Review Process

Several limitations should be acknowledged regarding the review process in the current systematic evaluation of laser and aPDT interventions for intraoral halitosis. First, despite restricting inclusion to randomized controlled trials to enhance methodological quality, substantial heterogeneity was identified across studies in terms of photosensitizers, laser parameters (such as wavelength, power, and duration), treatment protocols, and outcome measures, limiting the ability to perform quantitative meta-analysis and complicating direct comparison of results. Second, language bias could potentially affect the comprehensiveness of this review since only studies published in English were considered, possibly excluding relevant research available in other languages. Third, most of the included trials reported relatively short-term follow-up periods, which restricts the assessment of long-term efficacy and durability of treatment outcomes. Additionally, reporting inconsistencies across studies, particularly in microbiological evaluation techniques and patient-centered outcomes, introduced a risk of selective reporting bias. Finally, the exclusion of grey literature and unpublished studies may have contributed to publication bias, further affecting the robustness and generalizability of the findings. These limitations highlight the need for future research utilizing standardized methodologies, consistent reporting practices, and extended follow-up durations to strengthen the clinical evidence base for laser and aPDT interventions in managing intraoral halitosis.

### 4.4. Implications for Practice, Policy, and Future Research

The findings of this systematic review underscore the potential of laser and aPDT as promising, minimally invasive treatments for intraoral halitosis. For clinical practice, adopting these therapies could significantly improve patient compliance and treatment outcomes by offering targeted microbial reduction with fewer adverse effects compared to traditional methods. However, variability in current treatment protocols necessitates standardized clinical guidelines to ensure consistent implementation. At a policy level, establishing uniform regulations and training requirements for dental professionals employing laser and aPDT interventions could enhance patient safety and treatment quality. Future research should prioritize large-scale, multicenter randomized controlled trials with extended follow-up periods, standardized outcome measurements, and robust microbial assessments. Investigating combination therapies, novel photosensitizers, and home-based PDT applications could further expand the therapeutic utility and accessibility of these technologies in routine dental care.

### 4.5. Research Gaps and Translational Outlook

Although the reviewed evidence supports the short-term efficacy of laser and aPDT for halitosis, critical questions remain regarding long-term outcomes, recolonization dynamics, and patient-specific protocols. Mechanistically, emerging approaches such as targeted delivery of photosensitizers via nanoparticles, use of dual-wavelength lasers, and biofilm-penetrating formulations offer new directions [26,67,68]. Novel photosensitizers derived from plant-based polyphenols, curcuminoids, or chlorophyll analogs are under investigation for their enhanced biocompatibility and selective activation profiles. Additionally, home-use LED-based PDT devices and integration of digital tongue imaging may democratize access to care and improve monitoring [67,68]. Combination strategies involving PDT with probiotics (e.g., Lactobacillus salivarius) are also being explored to reduce recolonization and modulate the oral microbiome long term. Future trials should evaluate these innovative modalities in head-to-head RCTs and real-world settings to define their translational utility.

## 5. Conclusions

This systematic review provides robust evidence supporting the efficacy of laser and antimicrobial photodynamic therapy (aPDT) as minimally invasive and effective modalities for managing intraoral halitosis. Both interventions consistently demonstrate significant reductions in volatile sulfur compound levels and halitosis-associated microbial loads, frequently surpassing traditional mechanical and chemical methods in short-term outcomes. Despite these promising findings, the heterogeneity observed across treatment protocols highlights the need for standardized methodologies, extended follow-up periods, and rigorous multicenter trials. Future research should focus on clarifying optimal therapeutic parameters, evaluating long-term effectiveness, and exploring combined and home-based treatment options to fully establish laser and aPDT therapies as reliable standards in clinical practice for intraoral halitosis management. Although both laser and LED-based antimicrobial photodynamic therapy have shown efficacy in reducing halitosis and volatile sulfur compound levels, no conclusive evidence supports the superiority of one over the other currently. Future studies directly comparing light sources using standardized protocols are needed to establish optimal treatment strategies.

## Figures and Tables

**Table 1:** Search syntax used in the study.

Source	Search Term	Filters	Number of Results
PubMed	((“Halitosis”[MeSH] OR halitosis OR “oral malodor” OR “intraoral halitosis”) AND ((“Lasers”[MeSH] OR laser OR lasers) OR (“Photochemotherapy”[MeSH] OR “photodynamic therapy” OR aPDT OR “antimicrobial photodynamic therapy”)) AND (elimination OR treatment OR management OR efficacy OR effectiveness))	2015–2025 RCT	13
Embase	(‘halitosis’/exp OR halitosis:ti,ab,kw OR ‘oral malodor’:ti,ab,kw OR ‘intraoral halitosis’:ti,ab,kw) AND ((‘laser’/exp OR laser:ti,ab,kw OR lasers:ti,ab,kw) OR (‘photochemotherapy’/exp OR ‘photodynamic therapy’:ti,ab,kw OR apdt:ti,ab,kw OR ‘antimicrobial photodynamic therapy’:ti,ab,kw)) AND (elimination:ti,ab,kw OR treatment:ti,ab,kw OR management:ti,ab,kw OR efficacy:ti,ab,kw OR effectiveness:ti,ab,kw)	2015–2025	77
Scopus	(TITLE-ABS-KEY(halitosis OR “oral malodor” OR “intraoral halitosis”)) AND (TITLE-ABS-KEY(laser OR lasers OR “photodynamic therapy” OR aPDT OR “antimicrobial photodynamic therapy”)) AND (TITLE-ABS-KEY(elimination OR treatment OR management OR efficacy OR effectiveness))	2015–2025	73
Cochrane	(halitosis OR “oral malodor” OR “intraoral halitosis”) AND (laser OR lasers OR “photodynamic therapy” OR aPDT OR “antimicrobial photodynamic therapy”) AND (elimination OR treatment OR management OR efficacy OR effectiveness)	2015–2025	37

**Table 2:** Results of quality assessment for the included studies.

Study	Randomization Process	Deviations From Intended Interventions	Missing Outcome Data	Measurement of the Outcome	Selection of the Reported Result	Overall Bias Judgment
Alshahrani et al., 2020 [41]	Some concerns	Low	Low	Low	Some concerns	Medium
Joseph et al., 2016 [42]	Low	Low	Low	Some concerns	Some concerns	Medium
Bruno et al., 2024 [43]	Low	Low	Low	Low	Low	Low
Ciarcia et al., 2019 [44]	Some concerns	Low	Low	Low	Low	Medium
Dereci et al., 2016 [45]	Low	Low	Low	Low	Low	Low
do Vale et al., 2021 [46]	Low	Low	Low	Low	Some concerns	Medium
Gonçalves et al., 2020 [47]	Some concerns	Low	Low	Low	Some concerns	Medium
Joseph et al., 2014 [48]	Low	Low	Low	Low	Some concerns	Low
Krespi et al., 2021 [49]	Some concerns	Low	Some concerns	Low	Some concerns	Medium
Lopes et al., 2014 [50]	Low	Low	Low	Low	Low	Low
Lopes et al., 2016 [51]	Some concerns	Low	Low	Low	Some concerns	Medium
da Mota et al., 2016 [52]	Low	Low	Low	Some concerns	Some concerns	Medium
Pinto et al., 2016 [53]	Low	Low	Low	Low	Some concerns	Medium
Romero et al., 2021 [54]	Low	Low	Low	Low	Low	Low

**Table 6:** A summary of the assessment methods used in each study and their objectivity.

Study	Assessment Method	Objectivity	Evidence Strength
Alshahrani et al., 2020 [41]	Gas Chromatography	High	Strong
do Vale et al., 2021 [46]	Gas Chromatography	High	Strong
Ciarcia et al., 2019 [44]	Gas Chromatography + Microbiology	High	Strong
Gonçalves et al., 2020 [47]	Gas Chromatography	High	Strong
Lopes et al., 2014 [50]	Gas Chromatography	High	Strong
Lopes et al., 2016 [51]	Gas Chromatography + sulfide monitor	High	Strong
Romero et al., 2021 [54]	Gas Chromatography	High	Strong
da Mota et al., 2016 [52]	Gas Chromatography	High	Strong
Dereci et al., 2016 [45]	Halimeter (sulfur monitor)	Medium	Moderate
Krespi et al., 2021 [49]	Organoleptic + Halimeter	Medium	Moderate
Bruno et al., 2024 [43]	Organoleptic + Breath Alert™	Medium	Moderate
Joseph et al., 2014 [48]	Microbiology + Questionnaire	Medium	Moderate
Pinto et al., 2016 [53]	Microbiology + Gas Chromatography	Medium	Moderate

**Table 7:** Characteristics of light source used.

Study	Laser Details
Alshahrani et al., 2020 [41]	Diode laser (660 nm), continuous mode, power density: 3527 mW/cm^2^, average radiant power: 100 mW, energy density: 317.43 J/cm^2^, beam spot size: 0.028 cm^2^.
Joseph et al., 2016 [42]	Diode laser (655 nm, 1 W, continuous wave), 60 s duration at 60 mW/cm^2^ intensity, methylene blue photosensitizer at 10 mg/mL concentration.
Bruno et al., 2024 [43]	Blue LED combined with 20% annatto-based dye (Bixa orellana extract).
Ciarcia et al., 2019 [44]	Red LED (660 nm), continuous wave, average radiant power: 400 mW, energy density: 93.5 J/cm^2^, radiant energy: 36 J, beam spot size: 0.38 cm^2^, irradiation duration: 90 s per point, 4 irradiated points.
Dereci et al., 2016 [45]	Er,Cr:YSGG laser (2.78 μm wavelength), 1.5 W, 30 Hz pulse rate, 11% air, 20% water, pulse duration: 140 µs, 360° firing tip (Waterlase MD, Biolase, Irvine, CA, USA).
do Vale et al., 2021 [46]	Diode laser (THERAPY XT-EC^®^, DMC, São Paulo, Brazil); 660 nm wavelength; 0.005% methylene blue photosensitizer; irradiation at 6 points.
Gonçalves et al., 2020 [47]	Blue-violet LED (395–480 nm), energy: 9.6 J, radiant energy: 6.37 J/cm^2^ per point, irradiated six points on tongue dorsum with 20 s duration per point, 20% annatto-based extract.
Joseph et al., 2014 [48]	Diode laser (655 nm, 1 W, continuous wave), 60 s duration at 60 mW/cm^2^ intensity, methylene blue photosensitizer at 10 mg/mL concentration.
Krespi et al., 2021 [49]	Er,Cr:YSGG solid-state laser with pulsed water specifically for targeting oral biofilms.
Lopes et al., 2014 [50]	THERAPY XT-ES™; 660 nm wavelength; 100 mW power; continuous wave; fluence of 320 J/cm^2^; irradiance of 3537 mW/cm^2^; 9 J per point; 90 s exposure per point.
Lopes et al., 2016 [51]	THERAPY XT-EC^®^ (DMC ABC Equipamentos Médicos e Odontológicos, SP, Brazil); red diode laser; 660 nm wavelength; 0.005% methylene blue photosensitizer; direct contact method; irradiation of 6 sites.
da Mota et al., 2016 [52]	THERAPY XT-EC^®^ (DMC ABC Equipamentos Médicos e Odontológicos, São Paulo, Brazil); red diode laser; 660 nm wavelength; 100 mW output power; fluence 320 J/cm^2^; irradiance 3537 mW/cm^2^; energy 9 J per point; direct contact; irradiation at 6 points.
Pinto et al., 2016 [53]	Red diode laser (660 nm); methylene blue photosensitizer; combined with scaling and root planing.
Romero et al., 2021 [54]	Diode laser (THERAPY XT-EC^®^, DMC, São Paulo, Brazil); 660 nm wavelength; 100 mW power; fluence 318 J/cm^2^; irradiance 3537 mW/cm^2^; energy 9 J per point; irradiations at 6 points.

aPDT—antimicrobial photodynamic therapy, LED—Light Emitting Diode, Er,Cr:YSGG—Erbium, Chromium-doped Yttrium Scandium Gallium Garnet, CW—Continuous Wave.

## Data Availability

Not applicable.

## Supplementary Materials

The following supporting information can be downloaded at: https://www.mdpi.com/article/10.3390/pharmaceutics17081046/s1, PRISMA 2020 Main Checklist.
